# Large-Group One-Session Treatment: Feasibility in Highly Height Fearful Individuals and Predictors of Outcome

**DOI:** 10.3389/fpsyg.2019.02411

**Published:** 2019-10-24

**Authors:** André Wannemueller, Piotr Gruszka, Sarah Chwalek, Sonja Fröhlich, Miriam Mulders, Svenja Schaumburg, Johanna Schöttes, Sonja Wiederhold, Jürgen Margraf

**Affiliations:** Mental Health Research and Treatment Center, Ruhr-Universität Bochum, Bochum, Germany

**Keywords:** exposure treatment, group treatment, one-session treatment, large-group one-session treatment, height phobia

## Abstract

**Objective:**

Exposure based large-group one-session treatments (LG-OSTs) proved feasible in different situational fears and showed promising short- and long-term outcomes. Based on prior LG-OST protocols we explored feasibility and effectiveness of an LG-OST protocol in four cohorts of individuals highly fearful of heights (*N* = 104). Moreover, we aimed to identify predictors of LG-OST outcome in order to provide individualized treatment recommendations in the future.

**Methods:**

Participants’ fear of heights was assessed at pre- and post-treatment as well as at 5 months follow-up using questionnaires and a behavioral approach test (BAT). Pre-treatment indices of negative emotional traits and positive mental health, the extent by which fear-evoking expectancies were violated during exposure, and post-treatment group perception processes were assessed in order to predict the outcome.

**Results:**

The LG-OST procedure proved feasible and effective in terms of both subjective and behavioral fear of heights. Post-treatment effects sizes of questionnaires assessing fear of heights ranged between *d* = 0.94 – 1.43. After the treatment, about half of the participants (49.5%) were able to ascend an aerial fire ladder up to a maximum of 30 m (vs. pre-treatment 17.3%). Follow up results showed the long-term stability of effects. Among psychological constructs, positive mental health and expectancy violation were the strongest predictors of LG-OST long-term outcome.

**Conclusion:**

We conclude that exposure based LG-OSTs are feasible, effective and very efficient compared to individual face-to-face settings. Thus, they represent very promising treatment alternatives for situational fears including fear of heights. Moreover, clinical research may benefit from LG-OST protocols as its high standardization may facilitate the search for mediators and moderators of exposure outcomes.

## Introduction

In Europe, the demand for cognitive behavioral treatment (CBT) due to common mental health problems is steadily increasing. However, access to CBT for patients is often difficult as both number as well as capacity of clinical professionals are limited. Consequently, patients often have to accept long-waiting periods, which bear the risk of avoidable “chronifications” of their respective disorder. Moreover, CBTs produce immense costs, for either the patient as a self-payer or the national health care systems. For example, according to the German Statutory Health Insurance Association in the year 2015 alone, the German public health care system spent 1.978 Bn. EUR for outpatient psychotherapy, which indicates a 35% increase compared to 2009 (GKV, 2017). Therefore, efforts to develop easily accessible CBT-based interventions that are more efficient in terms of time- and cost-related aspects than already existing ones are urgently required. This might be particularly promising in disorders that are frequent, comparatively less impairing, and for which well-evaluated treatment strategies already exist, as is the case in phobic disorders.

With reported 12-month prevalence rates between 7 and 9% in western countries, Specific Phobias (SPs) are among the most frequent single mental disorders in adults (Wittchen et al., 2011). In fact, amongst women they are the most common single mental disorder (Regier et al., 1998). Depending on their respective contents, phobic fears are likely to differ in terms of how severe they impair the lives of affected individuals. However, at least during the worst episodes, great subjective distress and significant functional impairment has been demonstrated to result from SPs (e.g., Wittchen et al., 1998; Essau et al., 2000). For example, more work loss days per year are caused by SP than by chronic heart- or lung diseases (Alonso et al., 2004). Moreover, given the early onset and high persistence of most SPs as well as their common comorbidity (Wardenaar et al., 2017), the economic relevance of SPs should not be underestimated. Notwithstanding, for phobias the lowest help-seeking rate amongst all anxiety disorders has been reported (Wittchen et al., 1998).

Heights are very common feared situations. Oosterink et al. (2009) report a prevalence rate of 30.8% concerning subjectively high fear in height-related situations. With point prevalences for height phobia ranging between 1.6 and 5% (Becker et al., 2007; Depla et al., 2008; Oosterink et al., 2009; Kapfhammer et al., 2015) it can be assumed that the same is true for the pathological form of fear of heights (FoH). Height phobia turned out to be highly predictive for developing further anxiety symptoms such as panic attacks, agoraphobic fears or other SPs (Kapfhammer et al., 2015) and therefore has to be considered rather impairing. However, only 28.5% of affected individuals seek professional help which is a relatively low rate even compared to other SPs (Depla et al., 2008). Besides increasing efficiency, lowering the threshold of treatment access might be especially useful. It may enable individuals suffering from height phobia-like symptoms to seek treatment despite their uncertainty about the necessity of professional treatment.

Exposure-based treatment strategies have been proven to reduce anxiety symptoms including phobic fear very effectively (for a review of findings see Wolitzky-Taylor et al., 2008). Remarkably, existing reports concerning the effectivity of exposure in height phobia are somewhat mixed compared to studies targeting other SPs. There are some studies (e.g., Bourque and Ladouceur, 1980; Emmelkamp et al., 2002) that report significant reductions of behavioral, cognitive and physiological FoH symptoms following exposure treatment. However, a more recent meta-analysis (Arroll et al., 2017) presents data suggesting that *in vivo* exposure for acrophobia is not very effective in the long-run. It has been discussed that FoH might be less accessible to exposure strategies. This is based on the evidence that conditioning processes play a smaller role in the etiology of height phobia compared to other phobic fears (Menzies and Clarke, 1995; Poulton et al., 1998).

In the field of SPs, treatment approaches that combine both effectiveness and efficiency – at least when taking sheer treatment duration into account – already exist in the form of so-called ‘one-session-treatments’ (OSTs). Typically, OSTs that were first introduced by Lars-Goran Öst (1989) last for only 3 h or less and originally consisted of elements of *in vivo* exposure and participant modeling. As of today, also cognitive and motivational aspects have been added (see Zlomke and Davis, 2008 for a review). So far, OSTs have been successfully applied in a wide range of phobic disorders, such as flight phobia (Öst et al., 1997a), spider phobia (Öst et al., 1991; Hellström and Öst, 1995), dental phobia (Haukebø et al., 2008) and agoraphobia (Öst et al., 2001).

However, the therapist to patient ratio of individual one-session formats is still a limiting factor in terms of treatment efficiency. Application of OSTs in group-settings therefore would improve the efficiency of OSTs. So far, three research reports exist where OSTs have been delivered in small group settings of three to eight individuals (Öst, 1996; Öst et al., 1997b; Götestam, 2002). They all targeted spider phobia and reported feasibility and substantial fear reductions resulting from the group approaches, especially when employing direct rather than indirect exposure strategies such as participant modeling.

Encouraged by the results of small-group OST approaches, Wannemueller et al. (2016, 2017, 2018) recently conducted three open feasibility trials where they applied one-session formats in large-group settings (LG-OSTs). Regardless if the treatments were applied in highly spider fearful (*N* = 78), highly dental fearful (*N* = 43) or highly blood-injury-injection fearful individuals (*N* = 40), LG-OSTs led to substantial short-term as well as long-term reductions of subjective and behavioral fear responses. However, LG-OSTs were not useful for all participants. For example, it could be shown that genetic variations of the serotonin transporter gene emerged to strongly moderate the long-term LG-OST outcome (see Wannemüller et al., 2018 for more details).

Such results demonstrate a possible benefit of LG-OST beyond clinical usefulness, i.e., its utility as a research instrument. Due to their high standardization, LG-OST protocols might be especially useful in order to identify mediators and moderators of treatment outcome. So far, drawing conclusions on treatment moderators is often hampered, because quite unstandardized treatment formats are applied in heterogeneous samples. Moreover, high efficiency of LG-OSTs in terms of recruitment-, cost- and time-related aspects, could enable a more direct and easier transfer of mechanistic findings from the laboratory into clinical contexts.

The present study pursues two aims. First, it aims to investigate whether LG-OSTs are feasible and useful treatment options for a wide range of situational fears. Therefore, based on prior LG-OST concepts we developed an exposure-based LG-OST protocol targeting severe FoH and applied it in four different cohorts of highly height fearful individuals (*N*_tot_ = 104). Despite the somewhat inconsistent reports in terms of exposure-effectivity in FoH, we hypothesized that highly standardized LG-OST would lead to substantial reductions in FoH, analogous to the results observed for the prior fear cohorts.

Our second aim was to identify possible predictors of LG-OST outcomes. Traits such as anxiety and depressiveness have already been identified to negatively influence the outcome of CBT (e.g., Muris et al., 1998) and we hypothesized that they would do so also in the case of LG-OST. Moreover, we aimed to find out whether positive mental health indices and social perception processes such as the perceived group climate or entitativity referring to the perceived interconnectivity of group members (Campbell, 1958) predict the outcome of LG-OST. As the violation of fear-inducing expectancies has been considered as one of the key mechanisms of exposure success (Craske et al., 2014) we hypothesized that this factor would also be a predictor of the LG-OST outcome.

## Materials and Methods

### Participants

Participants for this feasibility trial were recruited via a local radio campaign, advertisements in newspapers and social networks. A website established for the project was open for registration for 3 months prior to the first treatment trial. The website contained information about prior LG-OST trials, the procedure of a treatment day and the team conducting the treatment. On this website, individuals could also specify their preferred treatment-date. Treatment was provided at four consecutive Saturdays between April and May 2018.

There were only three inclusion criteria: being of legal age (18 years) and reporting high subjective fear in and avoidance of height-related situations, such as approaching a shoulder of rock or looking down from a high bridge. Prior to registration, participants could screen their level of FoH and check if they were eligible for participation by completing an online-version of the “Anxiety”-subscale (20 items) of the Acrophobia Questionnaire (AQ; Cohen, 1977; see the measures section for a detailed description). We restrained from defining a certain score individuals had to exceed in the AQ in order to participate in the LG-OST. Rather, we recommended an individual to undergo the LG-OST procedure when he or she perceived fear in at least two of the described situations.

Altogether there were 104 participants, all Caucasian (64.1% female) with a mean age of 41.76 (*SD* = 12.56) years who appeared at the main municipal fire station of the city of Bochum where we conducted the LG-OST. They gave their informed consent to attend the LG-OST and having their data used for research purposes. At day 1 (14th April), *n* = 27 participants attended the treatment, at day 2 (21th April) *n* = 32, at day 3 (28th April) there were *n* = 24 and at day 4 (5th May) *n* = 21 participants (for a detailed comparison of the four cohorts see Supplementary Materials).

The local Ethics Committee of the Faculty of Psychology where the study was conducted approved the study.

### Procedure

A well-trained postgraduate clinical psychologist and licensed psychotherapist with vast clinical experience in the treatment of SPs performed the LG-OST. He was supported by another clinical psychologist with Master degree. They both supervised four Students all with Bachelor degrees in relevant exposure techniques so that they could support them in conducting the exposure exercises on the day of training. LG-OST was preceded and followed instantly by a behavioral approach test (BAT). During the BAT, participants could ascend with a turntable ladder up to a maximum of 30 m. Moreover, they completed a set of questionnaires assessing subjective components of FoH pre- and post-intervention as well as personality traits and questionnaires concerning social group perception. Before leaving, all participants were asked to voluntarily sign-up and to be available for FU-measures. After 5 months (*M* = 173.8 days; *SD* = 15.31), we invited them for a FU-assessment at the fire station of Bochum consisting of the same questionnaires and the BAT. Furthermore, we asked the participants how often they exposed themselves to FoH relevant situations within the post-treatment to follow-up interval and whether their fear-evoking expectancies had changed (0 = not at all to 4 = extremely). Altogether sixty participants (57.7%) participated at the FU-assessment.

### Large-Group One-Session Treatment (LG-OST)

The LG-OST was delivered to the patients in an auditorium at the main municipal fire station of the city of Bochum, Germany where the study was conducted and lasted for about 3 h. It consisted of three phases: a psychoeducation phase, a training phase and an exposure phase. Training materials (in German language) are available by request.

#### Psychoeducation Phase (About 30 min)

After entering the auditorium, participants watched a 12-min movie-clip, which included extensive information about heights and possible fear responses in height-related situations. In this clip, a firefighter specialized in high angle rescue explained how people typically respond to great heights and targeted frequently asked questions, such as the probability to faint or involuntarily jump down from ledges due to uncontrollable fear responses.

Afterward, the psychotherapist introduced some common ways of acquiring severe FoH. Based on the three-level approach (Lang, 1979) he explained the nature and utility of fear and its cognitive, behavioral and physiological consequences. Then he outlined the main aim of the following exposure phase i.e., gathering experiences that were incongruent to fear-evoking catastrophic expectancies. By the use of fear curves the therapist explained the difference between fear courses often anticipated by anxious individuals (staying at a constant fear level of 100% or even displaying a fear increase beyond 100%) and the course of fear that actually will occur. In order to demonstrate this he used the example of having dinner in a restaurant located in a lookout tower. At the very end of the psychoeducation phase, he explained that the use of a relaxed breathing technique could markedly decrease sympathetic activity and reduce stress and tension during the imminent exposure exercises (Busch et al., 2012).

#### Training Phase (About 20 min)

During the training phase, the psychotherapist instructed the participants to concentrate on deep, calm exhalation. He encouraged them to apply this deep breathing technique during the following exposure exercises in order to deal with upcoming fear responses. To reduce the risk of a possible misuse of the strategy as a safety behavior he instructed the participants very exactly and pointed out that the application of the technique is not a necessary means to cope with the fear reaction, but that the application can be helpful in order to dampen quite violent fear peaks, which are absolutely harmless for the organism even without the application of the breathing strategy. The exercise was described in more detail in Wannemueller et al. (2017).

#### Exposure Phase (About 120 min)

Exposure consisted of four steps: pictorial exposure with height-relevant pictures, video clips and two *in vivo* exposure elements i.e., exposure on a hose-drying tower and a high escape gallery. Prior to each exposure step, participants were asked to write down their expectancies concerning their own cognitive, bodily and behavioral responses in the respective situation. Moreover, they were asked to rate their expected achievement during the exposure exercises, for example, which height they expect to reach and what they believe could be the worst outcome such as that the railing might collapse when leaning on.

During pictorial exposure, five height-relevant pictures were presented. They all stemmed from an open access picture gallery depicting a person in height-relevant situations, such as sitting at the edge of the roof of a skyscraper. Pictures were presented for 60 s each and participants were asked to apply their breathing technique in case of displaying strong bodily fear responses. After exposure to the pictures, participants watched three video-clips. In the first two clips, people were filmed or filmed themselves in height-related situations (e.g., climbing up a crane). The third video consisted of a roller coaster ride filmed from the perspective of a participant’s head camera. Subsequent live-exposure was conducted in a hose-drying tower. The tower was equipped with five balconies with transparent grid floors at the height of 3, 6, 9, 12, and 15 m. Moreover, within the tower there was a duct to hang out the fire-hoses with a platform at about 20 m. Participants were asked to choose a height as an individual starting point for their exposure exercises according to the level of fear they could only just tolerate. At each balcony, a team member asked them to perform several exercises such as bending over the railing, reading some letters placed on the ground or jumping up. Participants could proceed to the next height-level whenever they felt to do so.

After 1 h of exposure, participants were asked to leave the tower and enter a free-hanging fire gallery in about 15 m. On the gallery, they could repeat the same exercises again as described for the tower exercise. They were supported by two licensed clinical psychologists in this exercise. After each exposure step, participants were asked to rate the level by which their respective fear-evoking expectancy has come true.

### Measures

#### Fear of Heights (FoH)

We used the *Acrophobia Questionnaire* (AQ; Cohen, 1977) to assess height-related anxiety (AQ-Anxiety) and avoidance (AQ-Avoidance). The AQ consists of 40 Likert-scaled items that ask participants to rate their anxiety and avoidance in 20 height-relevant situations (e.g., “riding a Ferris wheel”). Quality of psychometric properties of the AQ is well proven (Baker et al., 1973; Bourque and Ladouceur, 1980). Across different cohorts reliability (Cronbach’s alpha) was adequate to excellent, ranging between α = 0.72 and 0.92 (average = 0.81) for the AQ-Anxiety and AQ-Avoidance subscales. We found good internal consistencies (Cronbach’s alpha) of α = 0.89 for the total AQ, α = 0.85 for the AQ-Anxiety and α = 0.82 for the AQ-Avoidance subscales at pre-treatment assessment. The *Attitudes Toward Heights Questionnaire* (ATHQ, Abelson and Curtis, 1989) was used to assess how participants feel about heights (e.g., “Good/Bad,” “Pleasant/Unpleasant”). The ATHQ consists of six semantic differentials with each contrasting two attitude variables (e.g., “Good/Bad”; “Safe/Dangerous”). People are asked to rate their attitudes on a scale ranging from 0 (which corresponds to the first adjective) to 10 (which corresponds to the second adjective). The ATHQ has been shown to be treatment sensitive (Coelho et al., 2006). In the current study, Cronbach’s alpha for the ATHQ was adequate with α = 0.72. The *Heights Interpretation Questionnaire* (HIQ, Steinman and Teachman, 2011) assesses the likelihood of negative interpretations in two height-related situations using eight items each. Cronbach’s alpha for the total HIQ was good with α = 0.86 (Situation 1: α = 0.83; Situation 2: α = 0.89). In order to assess danger and anxiety expectancies in height-related situations we used the *Danger and Anxiety Expectancy Scales* for heights (DES-AES, Gursky and Reiss, 1987). For the DES Cronbach’s alpha was α = 0.88, for the AES α = 0.84 which indicates good internal consistencies. In order to be able to compare the results across different LG-OSTs, we applied the same 1-item screening as applied in our previous studies. It consists of the question *“How fearful are you when thinking about your personally most significant situation in regard to heights?”* (0 = not at all fearful; 10 = extremely fearful). A *Behavioral Approach Test* (BAT) was performed with the help of firefighters. At pre- and post-treatment assessment as well as at follow-up, participants were asked to enter the basket of a turntable ladder and ascend to a maximum of 30 m. A firefighter located in the basket secured the participants with a rope. During the climb, he was instructed not to talk to or calm the participants. Participants were asked to rate their subjective fear and if they would like to continue ascending each 3 m via a radio set. They could stop at any time and were asked to give a final fear rating at that point. Another firefighter operated the turntable ladder and monitored the procedure.

#### Negative Emotional Traits

The German version of the *State-Trait Anxiety Inventory* (STAI, Laux et al., 1981) consists of two subscales, each describing emotional states in 20 statements at present (state scale) and during the last 2 weeks (trait scale). Scores range from 20 (no anxiety) to 80 (high anxiety). Whereas the state-scale is highly sensitive for change, the trait-scale has a high retest reliability (*r*_tt_ = 0.96). In our sample, the internal consistency was excellent with Cronbach’s α = 0.90 for the trait-scale and α = 0.95 for the state-scale. We used the German version of the *Depression Anxiety Stress Scale-21* (DASS-21; Lovibond and Lovibond, 1995) to assess negative emotional states. The DASS-21 is a self-report questionnaire. Responses to each item are rated on a 4-point scale (0 = never; 3 = almost always). The DASS provides three separate scales, each consisting of a seven-item set: depression, anxiety and stress. The authors of the DASS report good to very good psychometric properties with internal consistencies (Cronbach’s alpha) of α = 0.91, α = 0.81, and α = 0.89 for the depression, anxiety and stress scale, respectively. In the present sample, we found an adequate to good internal consistency (Cronbach’s alpha) of α = 0.83 for the depression scale, α = 0.76 for the anxiety scale and α = 0.89 for the stress scale.

#### Positive Mental Health

In order to assess psychological and emotional aspects of well-being, we used the German version of the *Positive Mental Health Scale* (PMH-S, Lukat et al., 2016). It consists of nine items (e.g., *“I feel that I am actually well equipped to deal with life and its difficulties”*) that are rated on a scale ranging from 0 (do not agree) to 3 (agree). According to the authors, the PMH-scale exhibited a unidimensional structure across different test-cohorts and displayed good convergent (correlations between *r* = 0.51 and *r* = 0.81 with other mental health measures) and discriminant (non-significant correlations with age and gender in the patient sample) validity indices. In the current sample the instrument showed good internal consistency (Cronbach’s alpha) with α = 0.88.

#### Violation of Expectancies

In order to capture a wide range of possible fear- evoking expectancies concerning height-related situations and concerning the participants’ bodily and behaviorally responses we asked them three questions prior to the video exposure and both *in vivo* exposure exercises each accompanied by an example:

*1. What do you think about the tower/the free-hanging fire gallery?* (e.g., *“It’s not sure if the balcony will withstand the weight of all of us.”*)
*2. What do you think about your response when watching the movies entering the tower/the free-hanging fire gallery?* (e.g., *“I will be stiff as a poker due to my fear”*).
*3. What do you think about how you will behave when watching the movies entering the tower/the free-hanging fire gallery?* (e.g., *“I won’t be able to go all the way to the railing”*).

After each exposure step participants were asked to rate whether their expectancies proved to be true (yes/no) and in case of a positive rating to also rate the percentage by which it proved to be true. For each participant we calculated an index of *“expectancy violation”* by dividing the total number of expressed expectancies by the number of expectancies rated as proved true, regardless of the respective percentage ratings.

#### Group Perception

We used the *Group Entitativity Measure-Group Therapy* (GEM-GP, Hornsey et al., 2012) to assess the level of perceived entitativity within the treatment cohort. The GEM is a non-verbal measure consisting of six picture-items that depict five circles each. Whereas the four outer-circles represent the group, the middle-circle represents the individual. Circles are arranged in different distances to each other. With ascending picture number the distance between the circles decreases. At picture four, the circles start to overlap each other and nearly completely overlap at picture six. Individuals are asked to rate which picture best represents their perceived self-group relation. Hence, entitativity ratings range between 1 (low entitativity) and six (high entitativity). The authors of the GEM-GP report good discriminant and convergent validity indices for the GEM-GP as well as sufficient reliability indices. The short form of the Group Climate Questionnaire (GCQ-S, MacKenzie, 1983, German translation by the authors) assesses the participant’s perception of the group atmosphere on three scales: engagement, avoidance and conflict. It consists of 12 items (e.g., *“The members tried to understand why they do the things they do, tried to reason it out.”*) rated on a 7-point Likert-scale (0 = not at all to 6 = very much/extremely) indicating the extent of agreement. The GCQ scales have been proven to exhibit a good internal consistency (e.g., Kivlighan and Goldfine, 1991). In the current sample, however, Cronbach’s alpha for the GCQ was questionable with α = 0.61.

#### Subjective Rating of Therapy Success

We assessed subjective treatment success using the Global Success Rating (GSR). The question “*in comparison to the beginning of the training I feel*…” has to be answered on a 7-item Likert-scale ranging from 1 = much worse to 7 = much better.

### Statistical Analysis

We used repeated measures (rm) MANOVAs containing eight height-fear measures, that is, the AQ (both subscales), ATHQ, HIQ (both subscales), DES, AES and the BAT in order to analyze cohort-effects in terms of pre-treatment fear and LG-OST outcome.^1^ In order to test whether drop-out was selective, we conducted the same comparisons with FU-completers and non-completers. Stability of LG-OST-effects was analyzed using a three (time) × seven (instruments) rmMANOVA of FU-completers. In order to identify potential outcome predictors we used the mean post-treatment and follow-up fear reduction percentage averaged across all height-fear measures as dependent variable and sociodemographic variables (age, sex, and education), negative emotional traits (DASS total score), positive mental health (PMH-scale), the index of expectancy violation and group perception (GEM-GP, GCQ) as predictors in a regression analysis. There was no abrupt discontinuation during treatment, therefore all analyses could be conducted as a completer analysis. However, between measures, *n* could slightly differ, that is, due to incorrectly completed questionnaires (e.g., when two or more alternatives were marked for single choice questions) or missing data. In order to provide the respective effect size we report within-group effect sizes using Cohen’s *d* formula based on pooled standard deviations (Cohen, 1988). The Statistical Package for the Social Sciences (SPSS, version 24.0) was used to analyze the data.

## Results

### Did the Four Large-Group Cohorts Differ in Terms of Pre-treatment Variables or Treatment Outcome?

The four LG-OST cohorts did not differ in terms of age, *F*(3,100) = 1.54, *p* = 0.21, educational level, *F*(3,99) = 0.77, *p* = 0.52, or sex distribution, χ*^2^*(3) = 5.17, *p* = 0.16. The same was true for negative emotional traits (DASS-total; *F*(3,98) = 0.73, *p* = 0.53, DASS-subscales: all *p* > 0.15; STAI-trait, *F*(3,99) = 0.43, *p* = 0.73). Moreover there was no difference in levels of state fear (STAI-State), *F*(3,97) = 0.11, *p* = 0.96. A MANOVA containing pre-treatment scores of all height-fear measures did not show a main effect of cohort, *F*(24,282) = 1.03, *p* = 0.42. Likewise, we did not observe a cohort effect in any of the single-measures (all *p* > 0.07).

The same non-significant results emerged in terms of post-treatment fear reduction. The rmMANOVA containing the eight height-fear related measures did neither show a significant main effect of group, *F*(3,97) = 1.51, *p* = 0.22, nor a significant group by time effect, *F*(3,97) = 0.79, *p* = 0.50. For a detailed score-overview of all four LG-OST cohorts, see Supplementary Tables S1–S4. Due to these findings, we pooled all four cohorts into one big sample of *N* = 104 individuals.

### Is LG-OST Effective in Reducing Height-Fear at Post-treatment Assessment?

Averaged across all height-fear relevant subjective and behavioral measures, the LG-OST led to a post-treatment fear reduction of 27.96%, which indicates a significant pre- to post-treatment effect, *F*(7, 700) = 121.09, *p* < 0.001, η^2^ = 0.55, in the rmMANOVA. For *post hoc* separate rmANOVAs results and effect sizes, see Table 1.

**TABLE 1:** Means, SDs and effect strengths (Cohen’s *d*) of pre- to post changes in measures assessing FoH in LG-OST-participants.

	**Large-group One-session training (*N* = 104)**					
	**N^1^**	**Pre**	**Post**	**Statistics (pre vs. post)**		
		***M (SD)***	***M (SD)***	***F***	***p***	***ES [95% CI]***
**Sample characteristics and (clinical) statemeasures**						
Age (years)		41.76 (12.56)	–	–	–	–
Acad. Education (y.)	103	13.79 (3.03)	–	–	–	–
STAI-state	98	43.23 (11.61)	33.92 (7.97)	–	<0.001	0.93 [0.51 – 1.35]
STAI-trait	103	40.64 (10.07)	–	–	–	–
DASS (total)		30.46 (21.42)	–	–	–	–
DASS-depression		6.86 (6.96)	–	–	–	–
DASS-anxiety		7.42 (7.14)	–	–	–	–
DASS-stress		16.14 (10.38)	–	–	–	-
PMH-S		19.50 (4.31)	–	–	–	–
**Height-fear measures (questionnaires)**						
AQ-Anxiety	102	56.23 (17.12)	33.96 (19.57)	187.16	<0.001	1.21 [0.76 – 1.66]
AQ-Avoidance	103	12.92 (5.67)	7.36 (5.96)	90.59	<0.001	0.96 [0.54 – 1.38]
HIQ (Situation 1)	103	23.34 (6.86)	14.20 (4.62)	208.27	<0.001	1.56 [1.12 – 2.01]
HIQ (Situation 2)	103	19.31 (7.98)	12.74 (4.68)	76.85	<0.001	1.00 [0.58 – 1.43]
ATHQ	103	44.38 (9.08)	29.65 (10.77)	246.23	<0.001	1.48 [1.04 – 1.92]
DES	103	16.47 (5.00)	11.17 (4.18)	114.40	<0.001	1.15 [0.73 – 1.57]
AES	103	35.53 (6.66)	27.05 (7.05)	127.97	<0.001	1.24 [0.82 – 1.66]
1-Item Screening^2^	76	8.51 (1.56)	6.50 (2.66)	41.76	<0.001	0.92 [0.45 – 1.46]
**Behavioral approach test**						
meters (max)		16.11 (8.78)	22.07 (9.06)	93.29	<0.001	0.67 [0.27 – 1.07]
SUD at max		78.53 (23.71)	63.57 (30.39)	35.56	<0.001	0.55 [0.16 – 0.94]
**Perceived group climate**						
GCQ-engagement		–	3.71 (0.83)	–	–	–
GCQ-avoidance		–	2.70 (0.99)	–	–	–
GCQ-conflict		–	1.46 (0.71)	–	–	–
Entitativity	100	–	3.79 (1.07)	–	–	–
**GSR**	98	–	6.00 (0.74)	–		

*STAI-S, state-trait anxiety inventory State-Scale; STAI-T, state-trait anxiety inventory trait-scale; DASS, depression anxiety stress scale; AQ = acrophobia questionnaire; HIQ, heights interpretation questionnaire; ATHQ, attitudes toward heights questionnaires; DES, danger expectancy scale; AES, anxiety expectancy scale; SUD, subjective units of distress; GSR, global success rating; GCQ, group climate questionnaire. ^1^N only provided if <104. ^2^Due to a technical problem the 1-item screening was not administered in cohort 1.*

In the post treatment-BAT, 51 individuals (49.5%) of the participants reached the max. height of 30 meter. At pre-treatment only 18 participants (17.3%) were able to do so.

### Are the Effects of LG-OST Stable Over Time?

Sixty LG-OST-participants (57.7% of the initially treated sample) were available for FU-measures. The mean time interval between post- and FU-assessment was 173.8 days (*SD* = 15.31). Participants who returned did not differ from those who did not (*n* = 44) with regard to age, *F*(1,103) = 0.19, *p* = 0.66, gender, χ*^2^*(1) = 1.76, *p* = 0.19 and years of academic education, *F* = (1,101) = 0.69, *p* = *0.41*. The same was true concerning pre-treatment fear levels (STAI-State: *F*(1,102) = 0.81, *p* = 0.37) and the applied measures assessing negative emotional traits, that is, STAI-trait, *F*(1,101) = 0.35, *p* = 0.56, and DASS, *F*(1,100) = 0.55, *p* = 0.46. Moreover, participants who returned did not differ from those who did not neither concerning any pre-treatment measure of fear of heights (questionnaire and BAT) nor concerning the pre- to post-treatment reduction of FoH (all *p* > 0.09).

As can be seen in Table 2, we observed strong to very strong treatment effects (Cohen’s *d*) at follow-up concerning all questionnaire measures as well as in terms of behavioral approach. In the follow-up BAT, 47.8% of the participants reached the maximum level of 30 m. Mean fear reduction averaged across all subjective and behavioral fear measures was 25.54% compared to pre-treatment fear. Hence, subjective and behavioral fear reductions at post- and follow-up were on the same level.

**TABLE 2:** Means, SDs and effect strengths (Cohen’s *d*) of post- to follow-up and pre- to follow-up changes in measures assessing FoH in LG-OST-participants.

	**Large-group One-session training (*N* = 60)**								
	**Pre**	**Post**	**FU**	**Statistics (post vs. FU)**			**Statistics (pre vs. FU)**		
	***M (SD)***	***M (SD)***	***M (SD)***	***F***	***p***	**ES^1^**	***F***	***p***	**ES [95% CI]**
**Sample characteristics and clinical data**									
STAI-S	41.88 (12.10)	32.72 (6.76)	35.02 (9.79)	3.22	0.08	0.27	15.71	<0.001	0.62 [0.10 – 1.15]
STAI-T	39.76 (10.10)	–	37.02 (9.78)	–	–	–	12.15	0.001	0.28 [–0.24 0.79]
DASS-total	29.04 (21.14)	–	20.82 (19.40)	–	–	–	17.98	<0.001	0.41 [–0.12 – 0.93]
Depression	6.84 (7.40)	–	4.64 (6.20)	–	–	–	12.84	0.001	0.32 [–0.19 – 0.84]
Anxiety	6.60 (6.92)	–	5.26 (6.68)	–	–	–	2.96	0.09	0.20 [–0.32 – 0.71]
Stress	15.36 (9.38)	–	11.16 (8.70)	–	–	–	16.10	<0.001	0.46 [–0.06 – 0.99]
**Height-fear measures**									
AQ (Anxiety)	57.73 (16.70)	34.90 (20.33)	35.41 (20.34)	0.08	0.78	<–0.10	70.52	<0.001	1.20 [0.62 – 1.78]
AQ (Avoidance)	13.55 (5.65)	7.16 (5.63)	7.29 (6.27)	0.01	0.92	<0.10	61.24	<0.001	1.05 [0.46 – 1.63]
HIQ (Situation 1)	24.27 (6.79)	14.51 (5.00)	15.93 (6.76)	2.95	0.09	0.24	65.94	<0.001	1.44 [0.85 – 2.03]
HIQ (Situation 2)	18.50 (8.15)	12.83 (4.95)	12.79 (6.68)	0.01	0.93	<0.10	28.82	<0.001	0.77 [0.22 – 1.31]
ATHQ	45.13 (8.41)	30.33 (11.29)	31.53 (12.29)	0.74	0.39	<–0.10	65.28	<0.001	1.29 [0.74 – 1.85]
DES	16.68 (4.59)	11.41 (4.35)	12.20 (4.88)	1.95	0.17	–0.17	39.52	<0.001	0.95 [0.41 – 1.48]
AES	36.13 (6.81)	27.34 (7.17)	26.38 (8.55)	0.97	0.33	0.13	72.98	<0.001	1.26 [0.69 – 1.84]
1-Item Screening^2^	8.56 (1.56)	6.44 (2.72)	6.54 (2.86)	0.06	0.81	<0.10	17.83	<0.001	0.88 [0.33 – 1.43]
**BAT**									
meters (max)	15.33 (8.52)	22.49 (8.74)	22.41 (9.12)	0.02	0.89	<0.10	35.10	<0.001	0.80 [0.20 – 1.40]
SUD fear at max	78.13 (21.04)	60.04 (29.65)	49.17 (31.64)	5.75	0.02	0.34[0.06–0.77]	37.21	<0.001	1.08 [0.46 – 1.70]
**GSR**	–	6.02 (0.73)	5.76 (1.04)	5.22	0.03	–0.29 [–0.65 (–0.07)]	–	–	–

*STAI-S, state-trait anxiety inventory state-scale; STAI-T, state-trait anxiety inventory trait-scale; DASS, depression anxiety stress scale; AQ, acrophobia questionnaire; HIQ, heights interpretation questionnaire; ATHQ, attitudes toward heights questionnaires; DES, danger expectancy Scale; AES, anxiety expectancy scale; BAT, behavioral approach test; SUD, subjective units of distress; GSR, global success rating. ^1^Confidence intervals not provided in case of non-significant results. ^2^The 1-Item Screening was completed only in 39 participants due to a technical problem, in all other questionnaires N equaled 60.*

### What Are Predictors for the LG-OST-Outcome?

**FIGURE 1:**
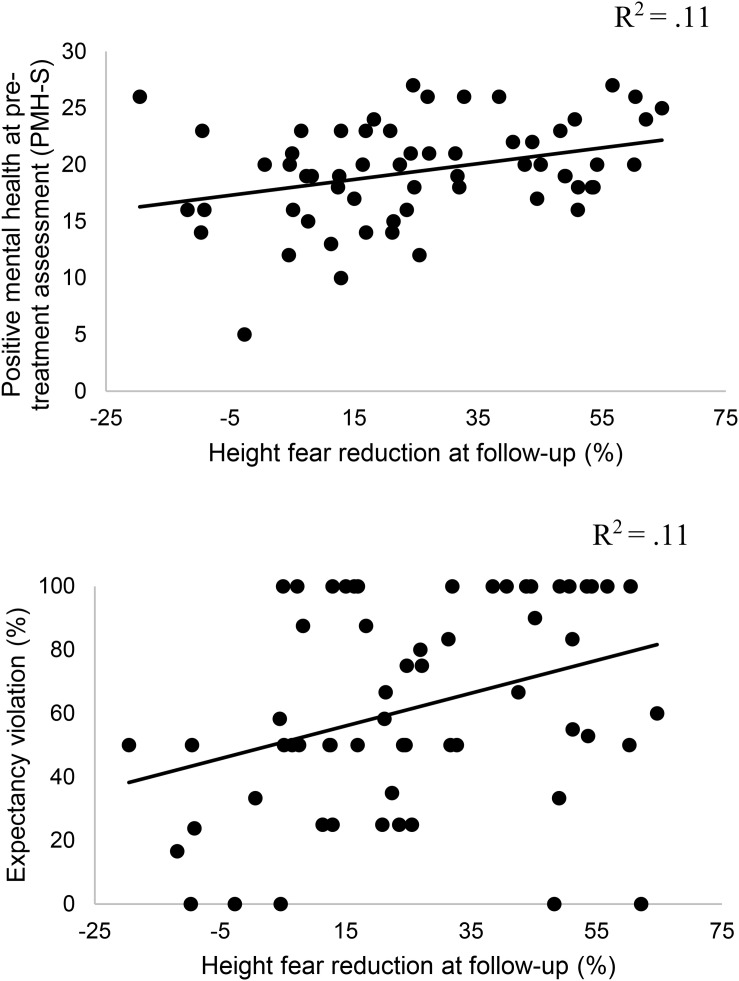
Correlations between pre-treatment positive mental health (PMH-S), expectancy violation and long-term reduction of fear of heights.

Concerning the prediction of the immediate fear reduction (within session habituation) operationalized by the percentage of pre-post differences across all measures concerning the FoH, multiple linear regression exhibited a good model equation, *F*(10,81) = 2.45, *p* = 0.013, *R*^2^_korr_ = 0.137, with female sex, β = 0.23, *p* = 0.029 and expectancy violation, β = 0.26, *p* = 0.014 being positive outcome predictors. An even better equation emerged concerning the prediction of long-term fear reduction (between session habituation), *F*(10,43) = 4.15, *p* < 0.001, *R*^2^_korr_ = 0.37. In this model, positive mental health (PMH-S), β = 0.40, *p* = 0.010 and again expectancy violation during treatment, β = 0.30, *p* = 0.018 emerged to strongly predict the long-term outcome, see also Figure 1. Moreover, educational level was a predictor of treatment outcome, β = 0.37, *p* = 0.004 with less years spent in academic institutions predicting a better outcome.

A separate analysis with follow-up data showed that the degree by which fear-evoking expectancies subjectively had changed in the post- to follow-up interval was associated with the long-term treatment outcome, also after controlling for gender, age, and educational level, *r*_*ab,c*_ = 0.56, *p* < 0.001.

## Discussion

We aimed to investigate feasibility and effectiveness of an exposure-based large-group one-session fear treatment (LG-OST) targeting severe FoH in this feasibility trial. In order to provide treatment recommendations in the future that best match individual needs, we further aimed to identify factors that predict the outcome of LG-OST.

Prior LG-OST trials suggest large-group exposure might represent promising treatment alternatives for situational fears, and proved especially promising in spider fear and blood-injury-injection fear (Wannemueller et al., 2016, 2018). Results of the present study provide further evidence for the suggestion also in terms of treating severe FoH.

Again, just like for the other LG-OST trials, in none of the four LG-OST cohorts we observed any signs of fear escalation or mass panic among the participants due to contagion effects at any time during the treatment. Overall, we have now applied exposure-based protocols in large group settings in cohorts with an *n* ranging between 21 and 79 seven times. Participants were affected by different situational fears and many of them showed heavy bodily and behavioral fear responses during the exposure exercises, which also was the case in the present height-fear LG-OST. However, oral reports of the participants suggest that observing other individuals who struggle with their fears has rather motivated them to overcome their own fear and avoidance tendencies. It facilitated them to overcome their resistance to take part in the exposure exercises. In an apparent contradiction to the participants’ reports, the group perception indices did not predict the outcome of LG-OST. However, these instruments primarily assess the interaction-quality between group members or the degree by which an individual perceives him-/herself to belong to a social group. In the case of LG-OST, the group members may rather represent a variety of coping models. Hence, sheer observational learning in its original sense (Bandura et al., 1969) rather than social group phenomena may explain the positive effect of the group format reported by the participants.

In terms of sheer LG-OST effectivity, results demonstrate that the applied protocol led to substantial and timely stable improvements in all measures targeting FoH. We observed the strongest effects concerning the subjective fear component and cognitive misconceptions with regard to heights. However, also in terms of reported avoidance and active approach behavior (BAT and SUD ratings) long-term effects were also large. This finding clearly contradicts the results of a recent meta-analysis (Arroll et al., 2017) that reported that *in vivo* exposure was not effective in the 1- to 9-months follow-up interval.

Based on the structure of LG-OST as well as the reported results one may speculate about possible explanations for successful between session habituation and long-term effectivity after exposure exercises targeting FoH. Albeit research is still in search for mechanisms underlying successful exposure treatments, inhibitory learning has been identified as a crucial factor (see Craske et al., 2014 for an overview). The concept of inhibitory learning represents the development of a secondary memory trace, in which the formerly fear associated situation (height) is no longer associated with the fear response but rather develops a fear inhibitory meaning (Bouton, 1993). According to Rescorla and Wagner (1972) the opportunity of learning increases as a function of mismatch between what is expected to occur and what actually occurs which is also called “prediction error”. Hence, exposure exercises should be designed in a way that they maximally violate fear-evoking expectancies. We aimed to realize this by consistently confronting the participants with their own fear expectancies and by asking them to rate the extent to which the exposure exercises matched their expectancies. Results demonstrate that short as well as long-term outcome was indeed strongly associated with changes in fear-evoking expectancies concerning height-related situations. Therefore, results not only underline the significance of expectancy violation as a mechanism underlying successful between-session habituation but also suggest future LG-OST trials to maintain expectancy violation as a treatment component.

The identification of treatment moderators may allow to provide evidence-based treatment recommendations in the future that optimally match individual needs. Interestingly, neither negative emotional traits at pre-treatment assessment nor group perception processes turned out to predict the outcome of LG-OST. Instead, among psychological constructs, only positive mental health indices were associated with beneficial LG-OST long-term effects. Positive mental health has been defined as an optimal way of psychological functioning and a general feeling of well-being and has been considered to represent more than the sheer absence of symptoms of a mental illness (Deci and Ryan, 2008). In outpatients receiving exposure therapy for anxiety disorders, pre-treatment positive mental health already turned out as a significant predictor for treatment outcome of multi session single treatments (Teismann et al., 2018). In our sample, we could not observe any associations between positive mental health and treatment-relevant behaviors such as the frequency of approaching height related situations in the follow-up interval. Future research should shed light upon explanatory factors for the observed association between positive mental health and exposure outcome that we observed for the LG-OST protocol. Interestingly, LG-OST proved especially effective in individuals with an academic education rather below average. Educational attainment repeatedly has been shown to interact with mental health status (Erickson et al., 2016), treatment-relevant behaviors such as premature drop-out (Roseborough et al., 2016) and exposure outcomes (Rizvi et al., 2009). It remains an open question why the LG-OST yielded those beneficial results in a group that other studies often have identified as a risk group concerning the outcome of CBT. Potentially, the one-session format of LG-OST makes premature drop-out more unlikely in these individuals or potentially they benefit from the very structured treatment delivery.

Several limitations of our study have to be considered. We applied the LG-OST-protocol in a group of highly height-fearful individuals who were not explicitly diagnosed with a specific phobia. This could limit the generalizability of our findings to phobic patient groups. However, pre-treatment indicators of FoH such as the AQ- or ATHQ-scores in our cohort were absolutely comparable to those reported for a cohort that met diagnostic criteria for a specific (acro-) phobia (Krijn et al., 2004). Future investigations of LG-OST effects in cohorts of individuals that meet all diagnostic criteria for a specific phobia would greatly inform future LG-OST protocols and may help to evaluate its clinical usefulness. Moreover, as customary for a feasibility-trial, we did not include an untreated, placebo-treatment or active control group condition. This could compromise the validity of findings, e.g., due to the lack of a waitlist- and/or a placebo control condition we cannot be sure that the observed changes in FoH were due to specific treatment components such as the exposure exercises or have to be attributed to rather unspecific factors such as participants’ expectation to be treated or even represent only a sheer effect of time. Future studies that randomly allocate participants to different control group conditions are needed in order to inform about which are the effective treatment components of LG-OSTs.

Furthermore, the number of LG-OST completers who returned for FU-assessment (62.5% in total) was relatively low. In order to realize the BAT again at follow-up assessment we depended on the help of firefighters. Therefore, follow-up assessments could only be realized at a few specific dates which might explain the relatively low response rate at follow-up assessment. Another concern that needs to be addressed is the incorporation of breathing exercises as means to prepare the participants for exposure as they could misuse this strategy as a safety behavior preventing the exposure to bodily fear symptoms. However, since in a large group setting, direct support from the therapists in case of heavy fear responses is more difficult to guarantee than in individual settings we aimed to provide our participants with more self-help strategies than we would have done within an individual treatment-setting.

In spite of the weaknesses mentioned, we conclude that an LG-OST targeting height-fear represents a very valuable treatment tool. Prior experience with LG-OSTs in spider-fear blood injury injection-fear and dental fear (Wannemueller et al., 2016, 2017, 2018) suggest that LG-OST might represent useful options either as a single treatment or as a step within a stepped care model of SP treatment. The size and stability of effects we observed here suggest that in case of FoH, the LG-OST protocol may sufficiently address the treatment needs of most participants – especially those who report good positive mental health indices prior treatment. A probable mechanism underlying positive LG-OST outcomes may consist in the violation of fear-evoking expectancies during the training and new forming of expectations that no longer are associated with threat.

## Data Availability Statement

The datasets generated for this study are available on request to the corresponding author.

## Ethics Statement

The studies involving human participants were reviewed and approved by the Ethics Committee of the Psychology Faculty of the Ruhr-Universität Bochum, Germany. The patients/participants provided their written informed consent to participate in this study.

## Author Contributions

AW, PG, SC, SF, MM, JS, and SW conceived and designed the study. AW analyzed the data. AW, SS, and JM wrote the manuscript.

## Conflict of Interest

The authors declare that the research was conducted in the absence of any commercial or financial relationships that could be construed as a potential conflict of interest.

## Footnotes

1 Due to a technical problem, the 1-Item measure was not applied at the day 1 cohort and therefore was excluded from the general rmMANOVA.
