# Supplementary material for: Large-Group One-Session Treatment: Feasibility in Highly Height Fearful Individuals and Predictors of Outcome
Source: Front Psychol. 2019 Oct 24;10:2411. doi: 10.3389/fpsyg.2019.02411 (PMC6842928; doi:10.3389/fpsyg.2019.02411)
Supplement: Supplementary file 1 [file Table_1.DOCX]

**Supplement**

**Table S1:** *Means, SDs and effect strengths (Cohen’s d) of pre- to post changes in measures assessing height fear in LG-OST-participants who attained the training at day 1.*

|  | **Large Group One-session training cohort 1 (N = 27)** | | | | | | | | | |  | | |
| --- | --- | --- | --- | --- | --- | --- | --- | --- | --- | --- | --- | --- | --- |
|  | | **Pre** | **Post** |  | | **Statistics (pre vs post)** | | | | |  |  |  |
|  | | ***M (SD)*** | ***M (SD)*** | ***F*** | | | ***p*** |  | ***ES [CI]*** | | | |  |
| ***Sample characteristics and clinical data*** | | | | | | |  |  | |  | |  |  |
| Age (years) | | 44.27 (11.77) | - | | - | | - | - | | | |  |  |
| Acad. Education (y.) (years) | | 13.19 (3.25) | - | | - | | - | - | | | |  |  |
| STAI-S | | 42.23 (6.71) | 33.73 (6.71) | | 20.81 | | <.001 | 1.27 [0.44 – 2.09] | | | |  |  |
| STAI-T | | 42.08 (10.07) | - | | - | | - | - | | | |  |  |
| DASS | | 17.38 (8.97) | - | | - | | - | - | | | |  |  |
| Depression | | 3.85 (3.28) | - | | - | | - | - | | | |  |  |
| Anxiety | | 4.52 (3.39) | - | | - | | - | - | | | |  |  |
| Stress | | 9.23 (4.00) | - | | - | | - | - | | | |  |  |
| ***Height-fear measures*** | | |  | |  | |  |  | |  | |  |  |
| AQ-Anxiety | | 56.95 (18.62) | 37.05 (21.73) | | 41.83 | | <.001 | 0.98 [0.19 – 1.78] | | | |  |  |
| AQ-Avoidance | | 13.79 (5.81) | 6.95 (7.05) | | 7.23 | | .013 | 0.66 [-0.13 – 1.42] | | | |  |  |
| HIQ (Situation 1) | | 23.69 (7.56) | 15.15 (4.68) | | 53.82 | | <.001 | 1.36 [0.52 – 2.20] | | | |  |  |
| HIQ (Situation 2) | | 20.80 (8.96) | 13.00 (4.89) | | 16.90 | | <.001 | 1.08 [0.27 – 1.89] | | | |  |  |
| ATHQ | | 47.64 (8.55) | 31.16 (10.75) | | 69.04 | | <.001 | 1.70 [0.82 – 2.58] | | | |  |  |
| DES | | 16.27 (5.97) | 11.23 (4.06) | | 19.44 | | <.001 | 0.99 [0.19 – 1.79] | | | |  |  |
| AES | | 35.42 (5.89) | 27.94 (6.64) | | 25.58 | | <.001 | 1.19 [0.37 – 2.01] | | | |  |  |
| 1-Item Screening | | - | - | | - | |  | - | | | |  |  |
| ***BAT*** | |  |  | |  | |  |  | |  | |  |  |
| meters (max) | | 14.03 (8.18) | 19.46 (9.44) | | 20.39 | | <.001 | 0.62 [-0.16 – 1.39] | | | |  |  |
| SUD fear at max | | 84.16 (21.64) | 76.68 (23.83) | | 4.18 | | n.s. | 0.52 [-0.25 – 1.29] | | | |  |  |
|  | |  |  | |  | |  |  | |  | |  |  |
| \| ***GSR*** \| - \| 5.93 (.60) - - \| - \| - \| \| --- \| --- \| --- \| --- \| --- \| | | | | | | | - |  | | - | |  |  |

***Note:*** STAI-S, State-Trait Anxiety Inventory State-Scale; STAI-T, State-Trait Anxiety Inventory Trait-Scale; DASS, Depression Anxiety Stress Scale; AQ = Acrophobia Questionnaire; HIQ, Heights Interpretation Questionnaire; ATHQ, Attitudes towards Heights Questionnaires; DES, Danger Expectancy Scale; AES, Anxiety Expectancy Scale; BAT, Behavioral Approach Test; SUD, Subjective units of distress; GSR, Global Success Rating;

**Table S2:** *Means, SDs and effect strengths (Cohen’s d) of pre- to post changes in measures assessing height fear in LG-OST-participants who attained the training at day 2.*

|  | **Large Group One-session training cohort 2 (N = 32)** | | | | | | | | | |  | |
| --- | --- | --- | --- | --- | --- | --- | --- | --- | --- | --- | --- | --- |
|  | | **Pre** | **Post** |  | | **Statistics (pre vs post)** | | | | |  |  |
|  | | ***M (SD)*** | ***M (SD)*** | ***F*** | | | ***p*** |  | ***ES [CI]*** | | |  |
| ***Sample characteristics and clinical data*** | | | | | | |  |  | |  | |  |
| Age (years) | | 43.44 (12.14) | - | | - | | - | - | | | |  |
| Acad. Education (y.) (years) | | 13.63 (3.21) | - | | - | | - | - | | | |  |
| STAI-S | | 42.18 (11.97) | 33.66 (7.63) | | 24.56 | | <.001 | 0.85 [0.13 – 1.57] | | | |  |
| STAI-T | | 40.53 (10.00) | - | | - | | - | - | | | |  |
| DASS | | 15.29 (12.64) | - | | - | | - | - | | | |  |
| Depression | | 3.44 (3.79) | - | | - | | - | - | | | |  |
| Anxiety | | 4.19 (4.42) | - | | - | | - | - | | | |  |
| Stress | | 7.78 (5.76) | - | | - | | - | - | | | |  |
| ***Height-fear measures*** | | |  | |  | |  |  | |  | |  |
| AQ-Anxiety | | 56.96 (18.26) | 38.00 (18.79) | | 49.83 | | <.001 | 1.02 [0.29 – 1.76] | | | |  |
| AQ-Avoidance | | 12.57 (4.95) | 8.20 (5.12) | | 28.36 | | <.001 | 0.89 [0.14 – 1.59] | | | |  |
| HIQ (Situation 1) | | 22.32 (6.83) | 14.84 (5.30) | | 40.32 | | <.001 | 1.22 [0.47 – 1.98] | | | |  |
| HIQ (Situation 2) | | 19.19 (7.89) | 13.38 (5.33) | | 29.12 | | <.001 | 0.86 [0.14 – 1.59] | | | |  |
| ATHQ | | 43.16 (8.72) | 30.23 (11.46) | | 60.18 | | <.001 | 1.27 [0.51 – 2.03] | | | |  |
| DES | | 16.28 (4.96) | 12.00 (4.41) | | 30.59 | | <.001 | 0.91 [0.18 – 1.64] | | | |  |
| AES | | 36.38 (7.13) | 28.09 (7.31) | | 34.81 | | <.001 | 1.15 [0.40 – 1.90] | | | |  |
| 1-Item Screening | | 8.25 (1.87) | 6.72 (2.68) | | 10.03 | | .003 | 0.66 [-0.05 – 1.37] | | | |  |
| ***BAT*** | |  |  | |  | |  |  | |  | |  |
| meters (max) | | 18.49 (8.69) | 23.34 (8.85) | | 31.89 | | <.001 | 0.55 [-0.15 – 1.26] | | | |  |
| SUD fear at max | | 75.78 (24.99) | 57.97 (33.48) | | 13.54 | | .001 | 0.60 [-0.11 – 1.31] | | | |  |
|  | |  |  | |  | |  |  | |  | |  |
| \| ***GSR*** \| - \| 5.87 (.81) - - \| - \| - \| \| --- \| --- \| --- \| --- \| --- \| | | | | | | | - |  | | - | |  |

***Note:*** STAI-S, State-Trait Anxiety Inventory State-Scale; STAI-T, State-Trait Anxiety Inventory Trait-Scale; DASS, Depression Anxiety Stress Scale; AQ = Acrophobia Questionnaire; HIQ, Heights Interpretation Questionnaire; ATHQ, Attitudes towards Heights Questionnaires; DES, Danger Expectancy Scale; AES, Anxiety Expectancy Scale; BAT, Behavioral Approach Test; SUD, Subjective units of distress; GSR, Global Success Rating;

**Table S3:** *Means, SDs and effect strengths (Cohen’s d) of pre- to post changes in measures assessing height fear in LG-OST-participants who attained the training at day 3.*

|  | **Large Group One-session training cohort 3 (N = 24)** | | | | | | | | | |  | |
| --- | --- | --- | --- | --- | --- | --- | --- | --- | --- | --- | --- | --- |
|  | | **Pre** | **Post** |  | | **Statistics (pre vs post)** | | | | |  |  |
|  | | ***M (SD)*** | ***M (SD)*** | ***F*** | | | ***p*** |  | ***ES [CI]*** | | |  |
| ***Sample characteristics and clinical data*** | | | | | | |  |  | |  | |  |
| Age (years) | | 37.50 (12.97) | - | | - | | - | - | | | |  |
| Education (years) | | 14.21 (2.84) | - | | - | | - | - | | | |  |
| STAI-S | | 43.88 (12.11) | 35.29 (10.71) | | 9.37 | | .006 | 0.75 [-0.08 – 1.58] | | | |  |
| STAI-T | | 38.88 (10.78) | - | | - | | - | - | | | |  |
| DASS | | 14.17 (10.45) | - | | - | | - | - | | | |  |
| Depression | | 2.96 (3.32) | - | | - | | - | - | | | |  |
| Anxiety | | 3.38 (3.16) | - | | - | | - | - | | | |  |
| Stress | | 7.83 (5.38) | - | | - | | - | - | | | |  |
| ***Height-fear measures*** | | |  | |  | |  |  | |  | |  |
| AQ-Anxiety | | 54.75 (17.57) | 27.17 (16.61) | | 51.41 | | <.001 | 1.61 [0.69 – 2.53] | | | |  |
| AQ-Avoidance | | 12.22 (5.70) | 4.70 (3.84) | | 34.01 | | <.001 | 1.55 [0.64 – 2.46] | | | |  |
| HIQ (Situation 1) | | 22.88 (7.13) | 12.38 (3.29) | | 53.86 | | <.001 | 1.89 [0.93 – 2.85] | | | |  |
| HIQ (Situation 2) | | 15.95 (5.09) | 11.50 (3.02) | | 11.98 | | <.001 | 1.06 [0.21 – 1.92] | | | |  |
| ATHQ | | 43.65 (8.35) | 28.00 (9.13) | | 67.62 | | .002 | 1.79 [0.84 – 2.74] | | | |  |
| DES | | 15.63 (5.22) | 9.54 (4.20) | | 25.35 | | <.001 | 1.29 [0.41 – 2.16] | | | |  |
| AES | | 34.75 (7.21) | 25.13 (6.92) | | 30.56 | | <.001 | 1.36 [0.47 – 2.25] | | | |  |
| 1-Item Screening | | 8.70 (1.06) | 5.87 (2.88) | | 19.13 | | <.001 | 1.30 [0.42 – 2.19] | | | |  |
| ***BAT*** | |  |  | |  | |  |  | |  | |  |
| meters (max) | | 15.46 (8.87) | 24.91 (8.26) | | 31.39 | | <.001 | 1.10 [0.24 – 1.96] | | | |  |
| SUD fear at max | | 72.26 (28.80) | 53.65 (30.36) | | 7.81 | | .011 | 0.63 [-0.19 – 1.45] | | | |  |
|  | |  |  | |  | |  |  | |  | |  |
| \| ***GSR*** \| - \| 6.33 (.70) - - \| - \| - \| \| --- \| --- \| --- \| --- \| --- \| | | | | | | | - |  | | - | |  |

***Note:*** STAI-S, State-Trait Anxiety Inventory State-Scale; STAI-T, State-Trait Anxiety Inventory Trait-Scale; DASS, Depression Anxiety Stress Scale; AQ = Acrophobia Questionnaire; HIQ, Heights Interpretation Questionnaire; ATHQ, Attitudes towards Heights Questionnaires; DES, Danger Expectancy Scale; AES, Anxiety Expectancy Scale; BAT, Behavioral Approach Test; SUD, Subjective units of distress; GSR, Global Success Rating;

**Table S4:** *Means, SDs and effect strengths (Cohen’s d) of pre- to post changes in measures assessing height fear in LG-OST-participants who attained the training at day 4.*

|  | **Large Group One-session training cohort 4 (N = 21)** | | | | | | | | | |  | |
| --- | --- | --- | --- | --- | --- | --- | --- | --- | --- | --- | --- | --- |
|  | | **Pre** | **Post** |  | | **Statistics (pre vs post)** | | | | |  |  |
|  | | ***M (SD)*** | ***M (SD)*** | ***F*** | | | ***p*** |  | ***ES [CI]*** | | |  |
| ***Sample characteristics and clinical data*** | | | | | | |  |  | |  | |  |
| Age (years) | | 40.89 (13.25) | - | | - | |  | - | | | |  |
| Education (years) | | 14.35 (2.66) |  | |  | |  |  | | | |  |
| STAI-S | | 44.10 (10.91) | 31.57 (5.37) | | 44.96 | | <.001 | 1.46 [0.50 – 2.42] | | | |  |
| STAI-T | | 41.05 (12.12) | - | |  | |  | - | | | |  |
| DASS | | 12.95 (10.03) | - | |  | |  |  | | | |  |
| Depression | | 3.43 (3.61) | - | | - | |  | - | | | |  |
| Anxiety | | 2.33 (2.50) | - | |  | |  | - | | | |  |
| Stress | | 7.19 (5.57) | - | |  | |  | - | | | |  |
| ***Height-fear measures*** | | |  | |  | |  |  | |  | |  |
| AQ-Anxiety | | 56.22 (13.87) | 33.11 (20.82) | | 54.59 | | <.001 | 1.31 [0.36 – 2.25] | | | |  |
| AQ-Avoidance | | 13.20 (6.68) | 6.95 (6.74) | | 51.95 | | <.001 | 0.93 [0.03 – 1.83] | | | |  |
| HIQ (Situation 1) | | 25.00 (5.68) | 14.15 (4.43) | | 81.25 | | <.001 | 2.13 [1.06 – 3.20] | | | |  |
| HIQ (Situation 2) | | 21.56 (8.80) | 12.78 (4.87) | | 25.99 | | <.001 | 1.24 [0.30 – 2.17] | | | |  |
| ATHQ | | 43.10 (10.59) | 28.81 (11.82) | | 49.14 | | <.001 | 1.27 [0.34 – 2.21] | | | |  |
| DES | | 17.86 (3.26) | 11.71 (3.68) | | 66.43 | | <.001 | 1.77 [0.76 – 2.78] | | | |  |
| AES | | 35.29 (6.53) | 26.57 (7.28) | | 40.16 | | <.001 | 1.26 [0.32 – 2.20] | | | |  |
| 1-Item Screening | | 8.71 (1.52) | 6.86 (2.35) | | 16.73 | | .001 | 0.94 [0.03 – 1.84] | | | |  |
| ***BAT*** | |  |  | |  | |  |  | |  | |  |
| meters (max) | | 15.84 (9.40) | 20.39 (9.06) | | 19.02 | | <.001 | 0.49 [-0.38 – 1.36] | | | |  |
| SUD fear at max | | 82.86 (15.86) | 67.38 (28.13) | | 12.91 | | .002 | 0.68 [-0.20 – 1.56] | | | |  |
|  | |  |  | |  | |  |  | |  | |  |
| \| ***GSR*** \| - \| 5.86 (.73) - - \| - \| - \| \| --- \| --- \| --- \| --- \| --- \| | | | | | | | - |  | | - | |  |

***Note:*** STAI-S, State-Trait Anxiety Inventory State-Scale; STAI-T, State-Trait Anxiety Inventory Trait-Scale; DASS, Depression Anxiety Stress Scale; AQ = Acrophobia Questionnaire; HIQ, Heights Interpretation Questionnaire; ATHQ, Attitudes towards Heights Questionnaires; DES, Danger Expectancy Scale; AES, Anxiety Expectancy Scale; BAT, Behavioral Approach Test; SUD, Subjective units of distress; GSR, Global Success Rating;
